# Performance Assessment of Biocompatible Metals Used in the Treatment of Femoral Neck Fractures

**DOI:** 10.1021/acsabm.2c00321

**Published:** 2022-06-08

**Authors:** Ferit Cakir, Fatih Mehmet Özkal, Ersin Sensoz

**Affiliations:** † Department of Civil Engineering, Gebze Technical University, 41400 Kocaeli, Turkey; ‡ Department of Civil Engineering, Atatürk University, 25240 Erzurum, Turkey; § Department of Orthopedics and Traumatology, Kartal Dr. Lütfü Kırdar Training and Research Hospital, 34865 İstanbul, Turkey

**Keywords:** femoral neck fractures, biocompatible metals, nonlinear finite-element analysis, cannulated screws in inverted triangle implants, performance decision

## Abstract

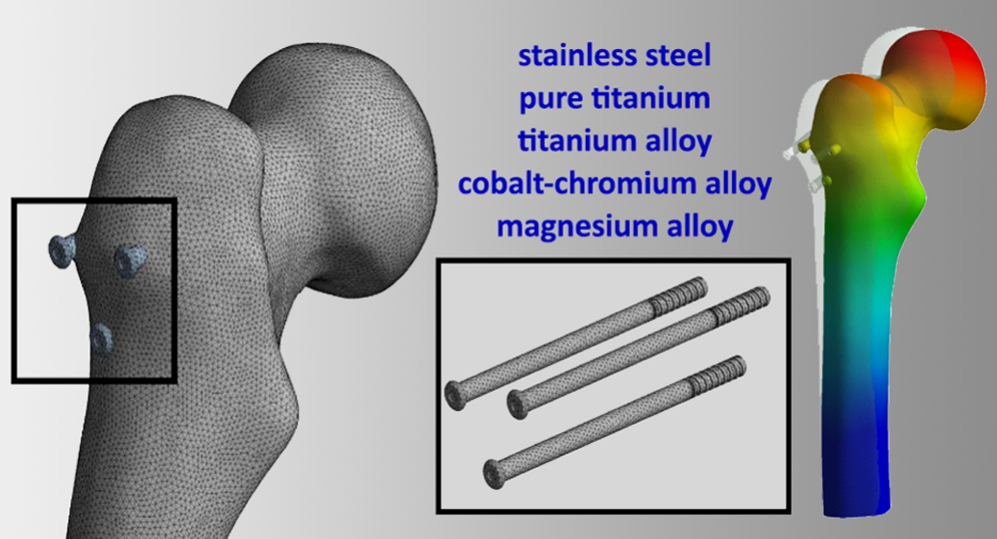

Femoral neck fractures (FNFs) are among the most common types of hip fractures. Particularly in young patients, these fractures require adequate fixation. These fractures, which are prevalent in elderly patients, are usually treated with implant applications. In implant applications, it is possible to find many different fixation configurations with various implant materials. The purpose of this study is to investigate the effects of metallic implant materials on fixation performance in the application of cannulated screws in an inverted triangle (CSIT), which are most preferred by orthopedic surgeons. Therefore, a femur bone with a type 2 fracture was numerically modeled and performances of CSIT implants with different biocompatible metals were investigated over nonlinear finite-element analyses (FEA). Within the study, stainless steel (SS), pure titanium (pTi), titanium alloy (Ti6Al4V), cobalt–chromium alloy (Co–Cr), and magnesium alloy (WE43) materials, frequently used as biocompatible implant materials, were taken into consideration and their performances were evaluated under static, vibration, and fatigue analyses. Throughout the comparison of analysis results and an optimality indicator formula, the optimum material was found to be the Co–Cr alloy on the basis of considered performance characteristics.

## 1 Introduction

Femoral neck fracture (FNFs) is a type of injury encountered in orthopedic patients. In particular, osteoporosis or low bone mass are among the most important causes of these fractures. The main aim of the treatment of an FNF is to minimize the trauma and return the patients to their prefracture functional level. While arthroplasty is preferred in the treatment of old patients, internal fixation precedes in younger patients.^1,2^ However, treatment of FNFs is a problematic and challenging issue for orthopedic surgeons, since poor or insufficient treatments might cause fault and catastrophic complications such as nonunion and avascular necrosis.^3,4^ Moreover, if the surgical intervention is inadequate or unsuccessful, patients could face some complications and discomfort situations. These risks should be considered and a convenient implant application for the fixation of FNFs should be preferred. Therefore, it is highly important to treat these fractures with ideal techniques and to choose the most appropriate method for treatment. The choice of treatment approach is performed mainly based on the fracture type, specific medical needs of the patient, and risk factors (e.g., lifestyle, nutrition, age, and sex). Today, internal implants are applied intensively for the treatment of FNFs. In implant applications, it is possible to find various fixation configurations with various implant materials.^5^ Regardless of the implant type, efficient usage of biocompatible materials is mandatory. Moreover, general anatomy, surgical approach, local healing rates in bone, effect of implant on bone, dynamic stress, weight-bearing capacity, and mechanical properties of implant materials are considered within the implant design. Especially, biocompatible materials to be used in the treatment of FNFs should be very durable and strong. For these reasons, biocompatible metals are the most commonly used implant materials since they have excellent strength, toughness, and wear resistance.

Major metal alloys have gained serious importance both biomedically and metallurgically today. When characteristics of biocompatible metals are examined, pure titanium and titanium alloy have a very important place among all implant materials used at present. However, it is possible to see that various materials other than titanium have recently been used as implant materials. Many studies have been conducted on the use of different materials in implants. Rony et al.^6^ focused on intraosseous metal implants by demonstrating that stainless steel (SS), cobalt (Co)-based alloy, titanium (Ti), and tantalum (Ta) are metals used in orthopedics. Moreover, some ceramics such as alumina or zirconia are used in orthopedic implants. Bandopadhyay et al.^7^ emphasized that stainless steel and cobalt chrome-based alloys were the first metallic materials that were successfully used in orthopedic applications because of their superior mechanical and anticorrosion properties. According to Hamidi et al.,^8^ although stainless steel has poor corrosion resistance and fatigue strength, it is still commonly used for nonpermanent implants such as internal fixation devices for fractures. Similarly, Madl et al.^9^ stated that titanium (Ti)-, cobalt (Co)-, and chromium (Cr)-based alloys have considerably replaced stainless steel in permanent implants. Willert et al.^10^ focused on fracture corrosion implanted with bone cement in femoral components prepared with Ti6Al7Nb and Ti6Al4V. In addition, Nakagawa et al.^11^ investigated the corrosion behavior of different titanium-based alloys in vitro due to the increase in palladium on the surface because of the increased pH values of the highly corrosion-resistant titanium-containing Pd’yi. Khan et al.^12,13^ conducted an accelerated corrosion test in vitro on CpTi, TiMo, and TiNbZr alloys and confirmed that Ti6Al4V and Ti6Al7Nb have the best wear and corrosion patterns.

The main purpose of this study was to investigate the mechanical performance and effects of the implant materials in the application of cannulated screws in an inverted triangle (CSIT), mostly preferred by orthopedic surgeons. For this reason, a femur bone with a type 2 fracture was numerically modeled and the performance of CSIT with different materials was investigated over finite-element analyses (FEA). Within the study, stainless steel (SS), pure titanium (pTi), titanium alloy (Ti6Al4V), cobalt–chromium alloy (Co–Cr), and magnesium alloy (WE43) materials, which are frequently used in implant applications, were taken into consideration and their performances were evaluated by static, vibration, and fatigue analyses via the finite-element method.

## 2 Materials and Methods

### 2.1 Cannulated Screws in Inverted Triangle (CSIT)

**Figure 1:**
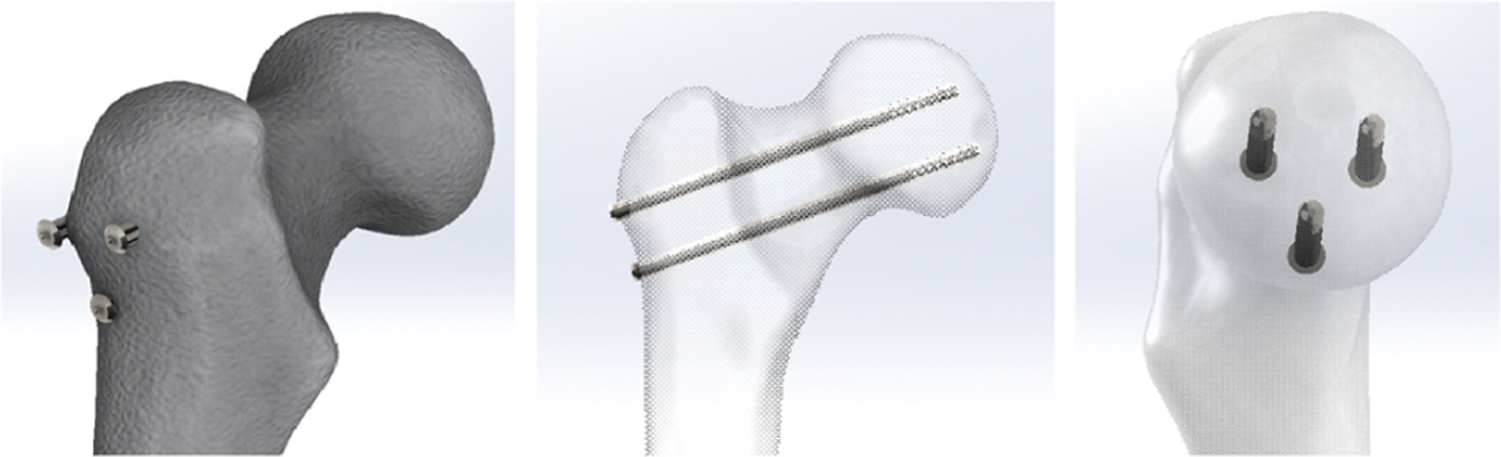
Cannulated screws in an inverted triangle (CSIT).

CSIT application is one of the most commonly used methods for the treatment of FNFs. The method is a minimally invasive method with percutaneous application after closed reduction, which shortens the duration of surgery and does not lead to bleeding. In this study, the position of the screws forms an inverted triangle and fractures are treated with three parallel screws, which have, ϕ7/length 90/20 mm terminally thread (Figure 1).

### 2.2 Biocompatible Materials

It is very important that materials to be used in implant applications must be biocompatible and behave in harmony with the bone tissue. For this reason, it is imperative to use materials that have been proven by credible scientific studies in practice. When previous studies are examined, it is understood that research on biocompatible materials is very limited and intensive studies are still required on this subject. This study focuses on five different materials that are applied for FNF treatment and have proven their reliability in terms of biocompatibility and bioactivity in the literature. These are stainless steel (SS), pure titanium (pTi), titanium alloy (Ti6Al4V), cobalt–chromium alloy (Co–Cr), and magnesium alloy (WE43).

In medical terms, “biocompatibility” describes the biological requirements for the use of a biomaterial. In other words, a biomaterial is biocompatible if it maintains cellular activity in the presence of molecular signaling systems without provoking or causing local or adverse effects in the host. Therefore, when a biocompatible material exhibits the expected beneficial tissue response and performs clinically relevant functions, it is considered biocompatible. Biocompatibility also includes cytotoxicity, genotoxicity, mutagenicity, carcinogenicity, and immunogenicity. An assessment of biocompatibility is normally performed using test animals, histological and pathological examinations of neighboring tissues, and host responses such as immunogenic, carcinogenic, and thrombogenic reactions. The materials used in this study are all classified as biocompatible materials in the literature and have been in use for many years.

Prior to the finite-element analysis (FEA), the engineering characteristics of the materials used in this study were investigated in detail.^14−19^ Within this context, previous studies in the literature were examined and mechanical properties of the materials were identified by considering these studies. The mechanical properties of the implant materials are shown in Table 1.

**Table 1:** Mechanical Properties of the Materials

	density (kg/m^3^)	yield/ultimate strength (MPa)	Young’s modulus (MPa)	Poisson’s ratio
Femur bone	550	tens: 135	15 000	0.30
		comp: 205		
316L Stainless steel (SS)	8000	190/490	193 000	0.27
Pure titanium (pTi)	4510	485/550	105 000	0.37
Titanium alloy (Ti6Al4V)	4430	795/860	104 800	0.31
Cobalt–chromium alloy (Co–Cr)	1000	520/790	243 000	0.29
Magnesium alloy (WE43)	1800	150/250	45 270	0.27

## 3 Finite-Element Analyses (FEA)

### 3.1 Numerical Modeling

**Figure 2:**
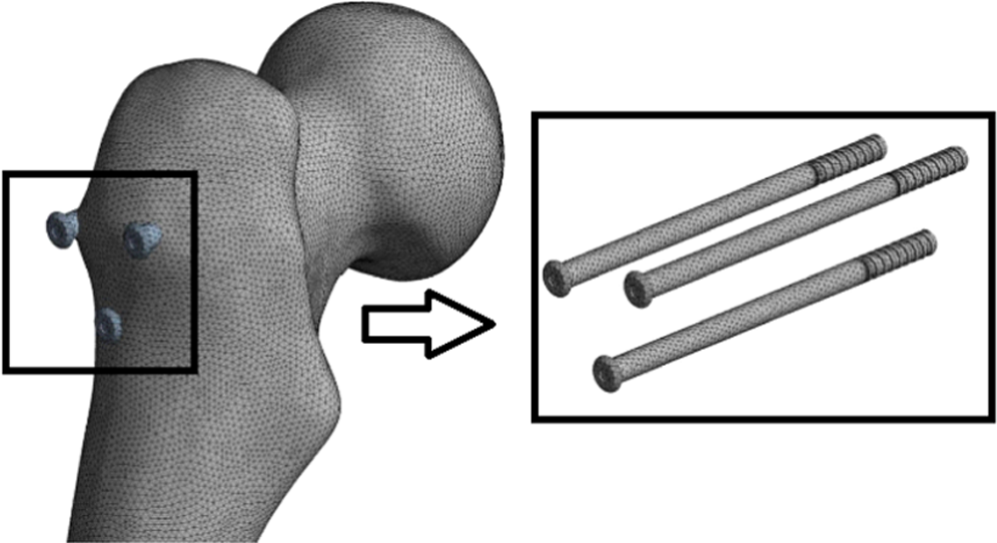
Numerical model of the CSIT.

In recent years, it has become common to use many different intellectual computer models together with the developing computer technologies and to develop solutions to different problems through numerical modeling techniques. Particularly, the number of computer-based studies using the finite-element method (FEM) has increased considerably.^20^ In the numerical modeling approach based on the finite-element method in this study, femur bone and internal fixation implants were modeled using a general-purpose finite-element software, ANSYS Workbench.^21^ Solid186 elements, which have 20 nodes and 3 degrees of freedom per node, were used and tetrahedral element shape was preferred. In numerical models, femur bone and implants were discretized with 409289 solid elements with the corresponding 612308 nodes for the CSIT (Figure 2).

Convergence of the mesh determines how many elements must be included in a model to make sure that the results of an analysis are not affected by changing its size. With the decreasing element size, the system response (stress, deformation) converges to a repeatable solution. In this study, it is verified through mesh convergence analysis that the FEA model reaches a correct solution using an iterative method. The response of interest is the maximum vertical deflection, and it is checked on the mesh size versus deflection and solution time by varying the number of elements along each edge. As a result, the mesh size and mesh quality used in the study were determined by this approach.

Among the analyses, five different biocompatible materials were handled one by one and the performances of these materials were evaluated by considering the same boundary conditions, same mesh numbers, and same loadings. For boundary conditions, all nodes located on the femur shaft were fixed against rotations and translations in all directions. In this research, a bonded connection was defined for the interfaces between screws and femur while the fracture surface on the femur was modeled as a friction surface with a 0.3 fraction coefficient.^22^ Moreover, all of the numerical models were subjected to a vertical displacement load (1 mm vertical displacement 7° valgus).

The femur has two axes, which are mechanical and anatomical. A mechanical axis is a line that passes through the bone to bear weight. An anatomic axis of the femur is the line connecting the midpoints of the tibia at the joint line and at the junction of the distal 1/4 and proximal 3/4 of the femur. Therefore, the mechanical axis of the femur is different from the anatomical axis. The valgus of the distal femur is determined based on the angle between the anatomical and mechanical axes of the femur. When a normal person is standing, the valgus angle is 7°. Therefore, within the scope of this study, analyses were carried out considering the 7° valgus angle. A similar situation has been used in many studies in the literature.^23−25^

### 3.2 Numerical Analyses

#### 3.2.1 Static Analyses

**Figure 3:**
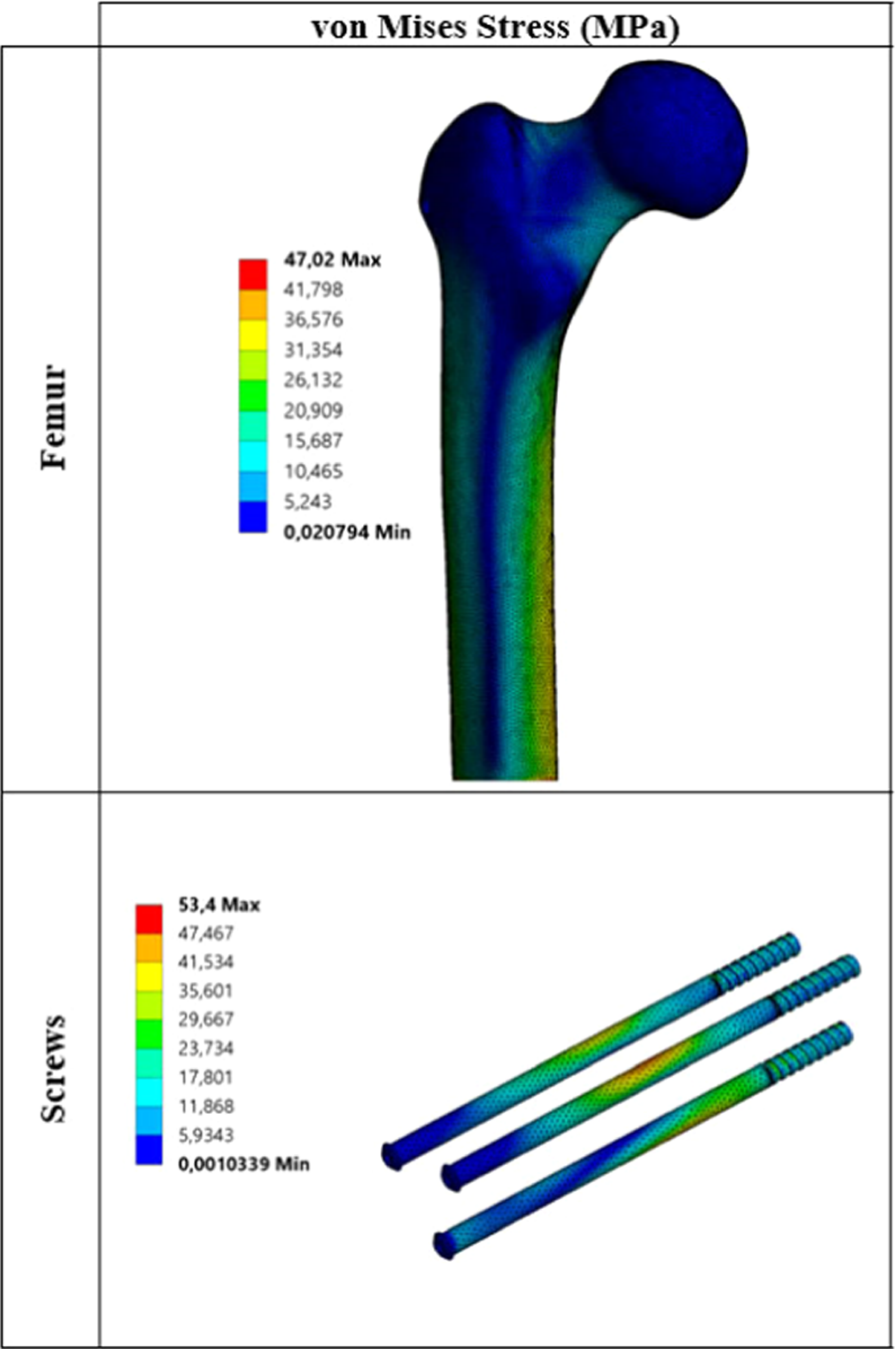
Critical stress distribution under static load (metal: stainless steel).

**Figure 4:**
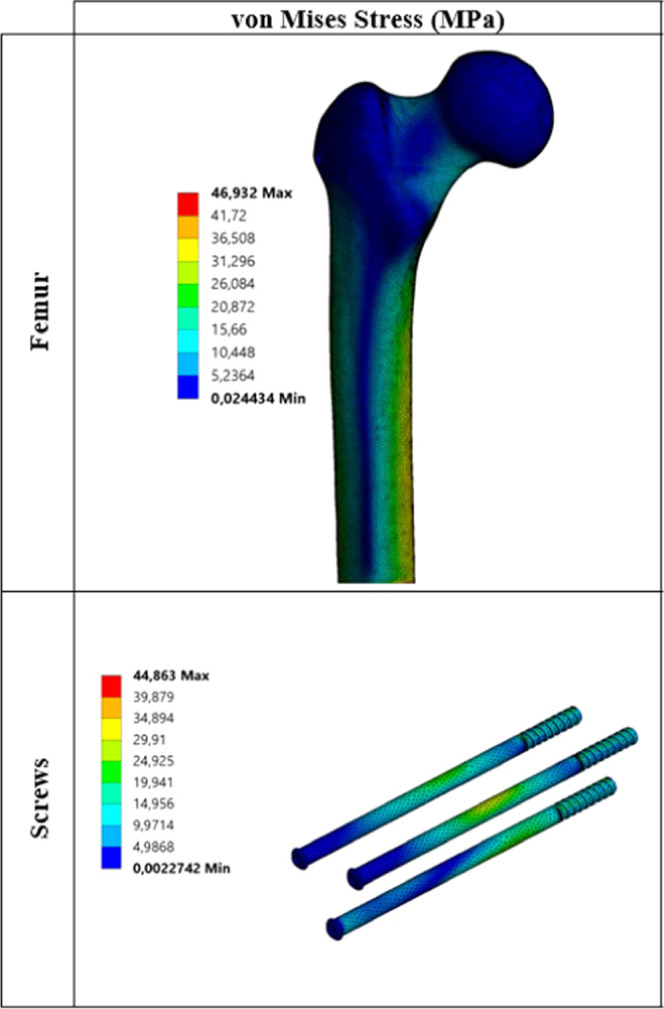
Critical stress distribution under static load (metal: pure titanium).

**Figure 5:**
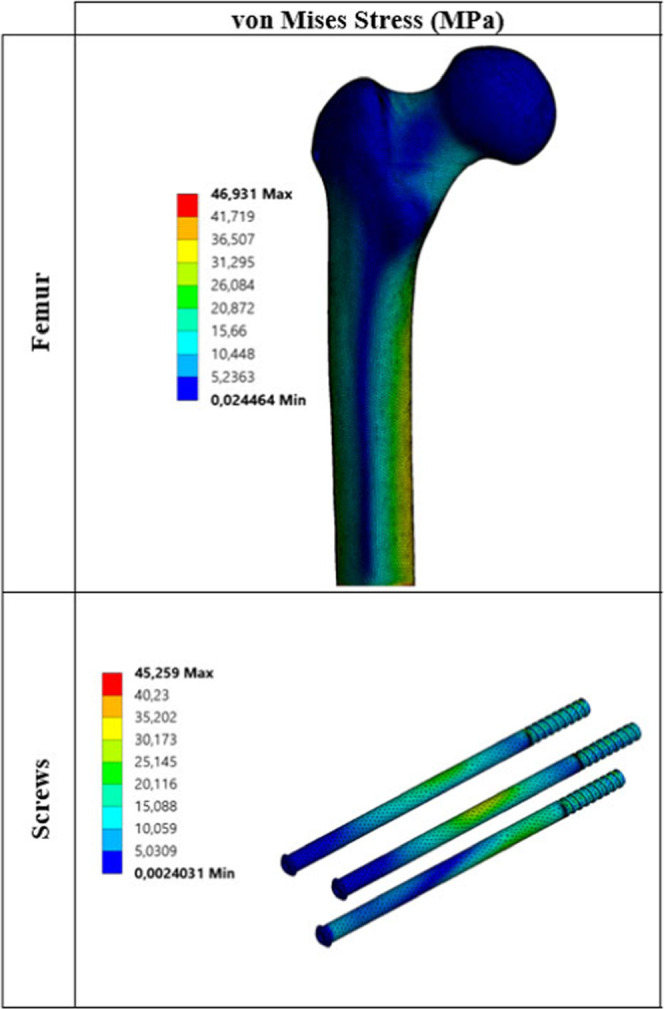
Critical stress distribution under static load (metal: titanium alloy).

**Figure 6:**
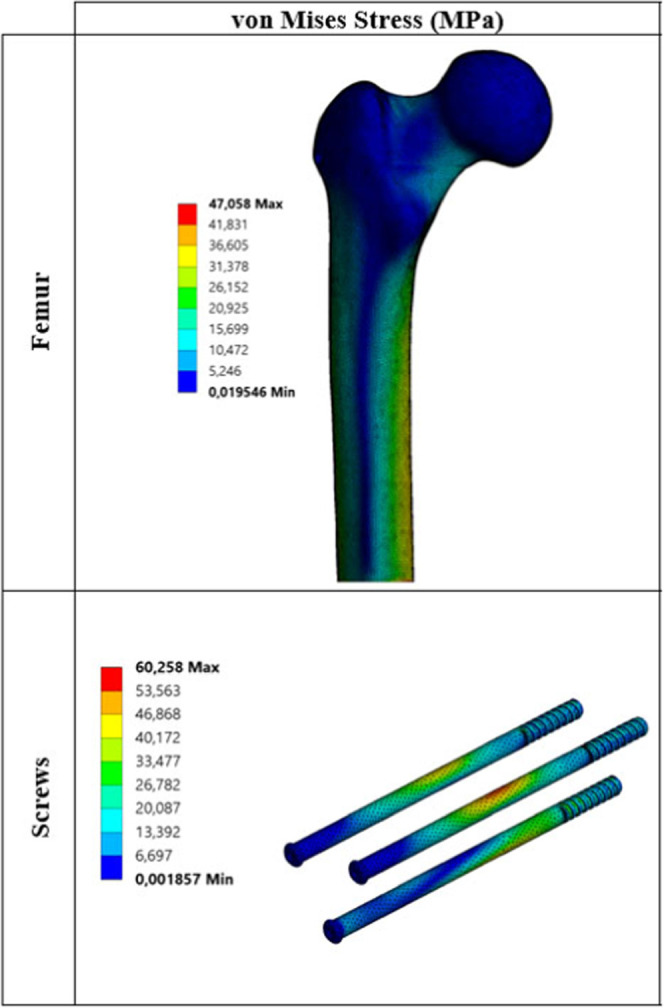
Critical stress distribution under static load (metal: cobalt–chromium alloy).

**Figure 7:**
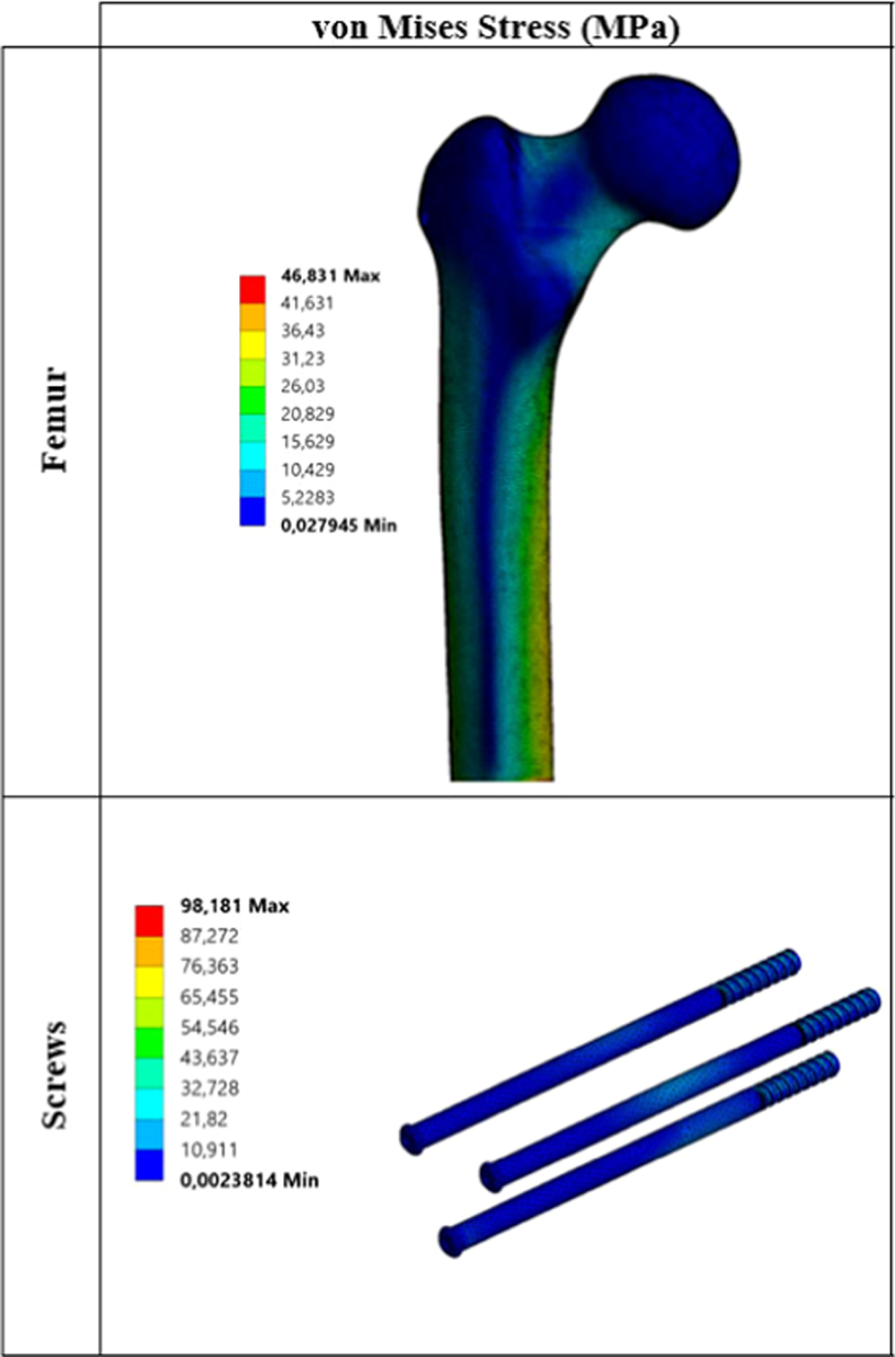
Critical stress distribution under static load (metal: magnesium alloy).

In the first step of the numerical analyses, static FEA was conducted using ANSYS Workbench^21^ taking a static load into consideration for each type of implant. In static analyses, the femur subjected to static forces was analyzed, and the critical stresses among each implant were calculated. Reaction forces were initially determined under static load (Table 2). After that, von Mises, maximum principal (tensile), minimum principal (compressive), and maximum shear stresses were determined for each material, respectively. Critical von Mises stress distributions under static load for each biocompatible metal are given in Figures 3–7.

**Table 2:** Calculated Reaction Forces under Static Load

material	reaction force (N)
SS	2009.7
pTi	2005.9
Ti6Al4V	2005.8
Co–Cr	2011.3
WE43	2001.2

#### 3.2.2 Vibration Analyses

**Figure 8:**
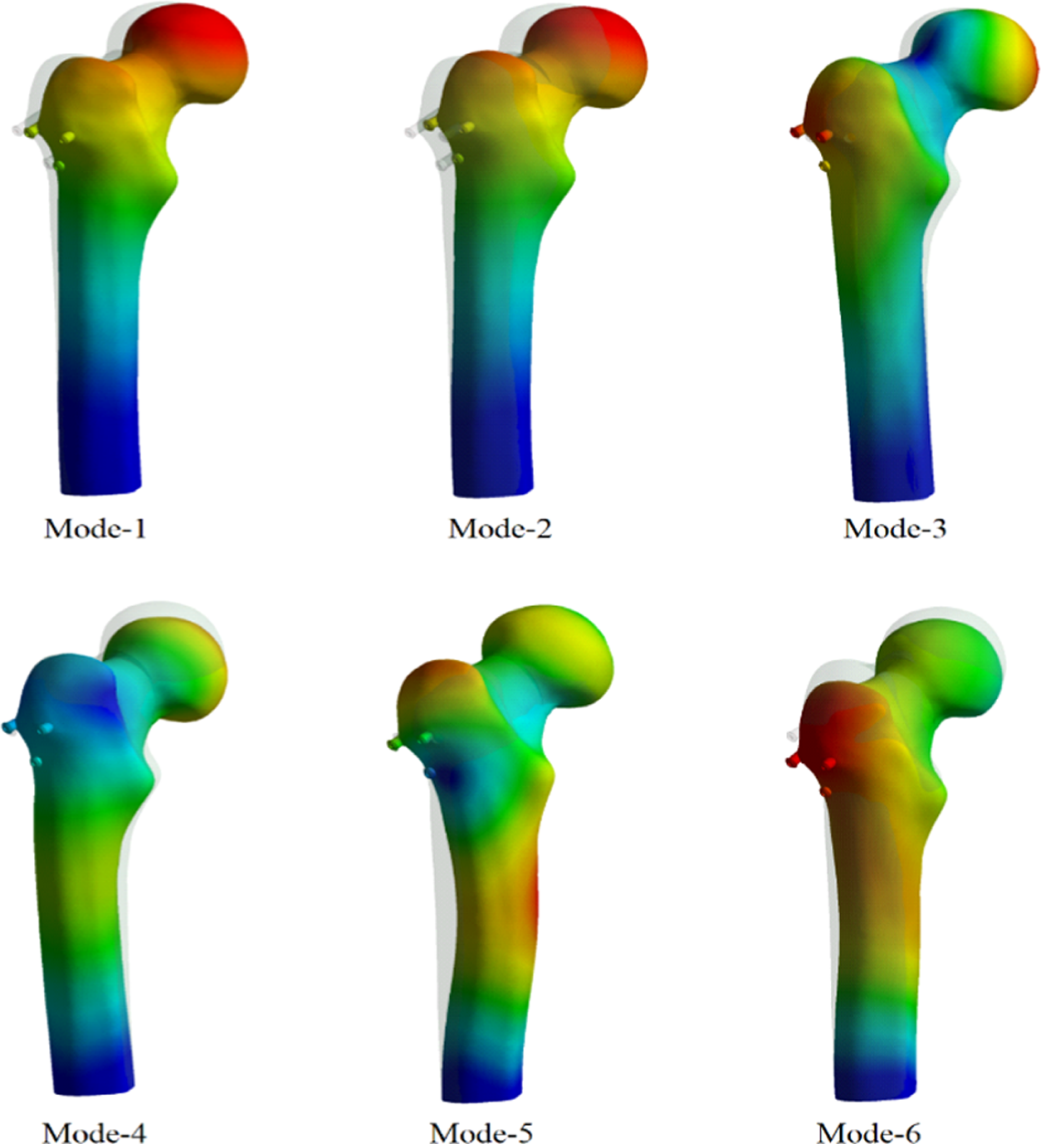
First six mode shapes for the vibration analyses.

Vibration analysis, which is also known as modal analysis, is preferred for the dynamic characterization of any physical structure. In this study, first, modal analysis was performed to observe the dynamic behavior of a femur treated with an implant. Frequency values obtained after the analyses are given in Table 3. When mode shapes were examined, it was observed that although the material properties were changed, mode shapes did not change and the dynamic behavior was the same. The first two modes were transverse modes, the third mode was torsional mode, and the others exhibited bending modes. The first six mode shapes achieved by the vibration analyses are given in Figure 8.

**Table 3:** Frequencies of the First Six Modes

	mode shape					
material	1	2	3	4	5	6
SS	266.2	278.8	1165.0	1842.2	2653.5	4035.0
pTi	272.8	290.9	1184.3	1906.3	2652.5	4231.3
Ti6Al4V	273.1	290.6	1181.6	1906.9	2655.0	4236.2
Co–Cr	314.0	328.3	1357.7	2021.1	2788.5	4477.5
WE43	269.1	294.7	1151.7	1950.0	2623.6	4407.8

#### 3.2.3 Fatigue Analyses

**Figure 9:**
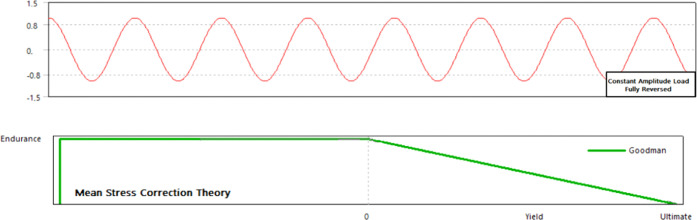
Periodic loading history used in the analyses.

**Figure 10:**
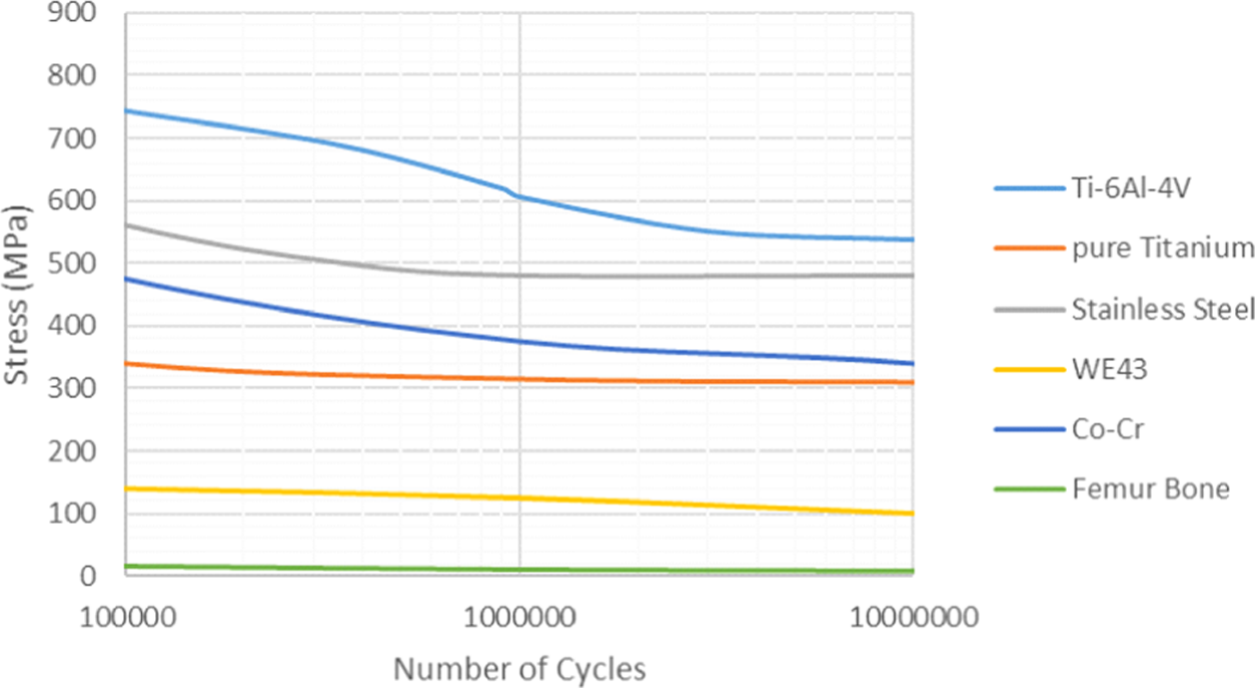
Stress–life (S–N) curves of the biocompatible metals.

Fatigue failures of implant metals are significantly important for the mechanical life of materials (Teoh 2000). Fatigue failures occur when the materials are subjected to repeated cycling loading and do not exhibit any plastic deformation. Therefore, it is necessary to determine the fatigue life of metallic materials. For this purpose, in this study, the fatigue strength and fatigue life of biocompatible metals were also investigated using numerical fatigue analyses. Among the fatigue analyses, biocompatible metals were subjected to a periodic and fully reversed loading at a cyclic vertical load of 1000 N. Moreover, the mean stress correction theory (Goodman Theory) was used in analyses (Figure 9) and equivalent (von Mises) stresses were investigated. The stress–life curve approach (S–N) was used, since this is a popular technique to determine the fatigue life of various metals. The stress–life curves used in the analyses are shown in Figure 10, and the determined fatigue lives of the biocompatible metals is given in Table 4.

**Table 4:** Determined Fatigue Lives of the Biocompatible Metals

	SS	pTi	Ti6Al4V	Co–Cr	WE43
Fatigue life cycles	4.85 × 10^6^	3.15 × 10^6^	6.20 × 10^6^	3.75 × 10^6^	1.25 × 10^6^

## 4 Evaluation of Numerical Results

Within the scope of the study, internal implants made from five different biocompatible metals were numerically modeled and their performances were investigated using finite-element analyses. These are static, vibration, and fatigue analyses that were carried out for each material. In this section, the results obtained from numerical analyses were examined and compared with each other.

The values obtained as a result of static analyses are divided into two main parts. These are critical stresses on the femur (Table 5) and critical stresses on the screws (Table 6). When changes in the stress on the femur under static loading are examined, stress results are generally close to each other. When critical stresses are examined, it is seen that maximum von Mises stress occurs for the Co–Cr material. The lowest von Mises stress is observed for pTi and Ti6Al4V materials. Similarly, when the maximum principal, minimum principal, and maximum shear stresses are examined, it is seen that stress distribution on the femur is not very different from von Mises stress distribution. Stresses on the screws are significantly different from the stresses on the femur. When different materials are taken into consideration, critical stresses on the femur do not show much difference from one material to another, whereas the situation with screws is quite different. For example, when von Mises stresses on the screws are examined, the lowest stress of 44.86 MPa is observed at pTi, while the maximum stress of 98.18 MPa is observed at WE43. As shown, von Mises stress at WE43 is about 2.20 times that at pTi. For other critical stresses, the situation is not different. Critical stresses for all cases reach maximum values in the WE43 material.

**Table 5:** Critical Stresses on the Femur Obtained by the Static Analyses

	max. von Mises stress (MPa)	max. principal stress (MPa)	min. principal stress (MPa)	max. shear stress (MPa)
SS	47.02	59.33	66.15	25.42
pTi	46.93	59.22	66.02	25.37
Ti6Al4V	46.93	59.22	66.02	25.37
Co–Cr	47.06	59.38	66.20	25.44
WE43	46.83	59.09	65.88	25.32

**Table 6:** Critical Stresses on the Screws Obtained by the Static Analyses

	max. von Mises stress (MPa)	max. principal stress (MPa)	min. principal stress (MPa)	max. shear stress (MPa)
SS	53.40	67.67	83.19	29.18
pTi	44.86	49.19	61.37	25.71
Ti6Al4V	45.26	47.13	60.56	26.04
Co–Cr	60.29	77.23	65.40	32.92
WE43	98.18	96.00	96.79	54.81

**Figure 11:**
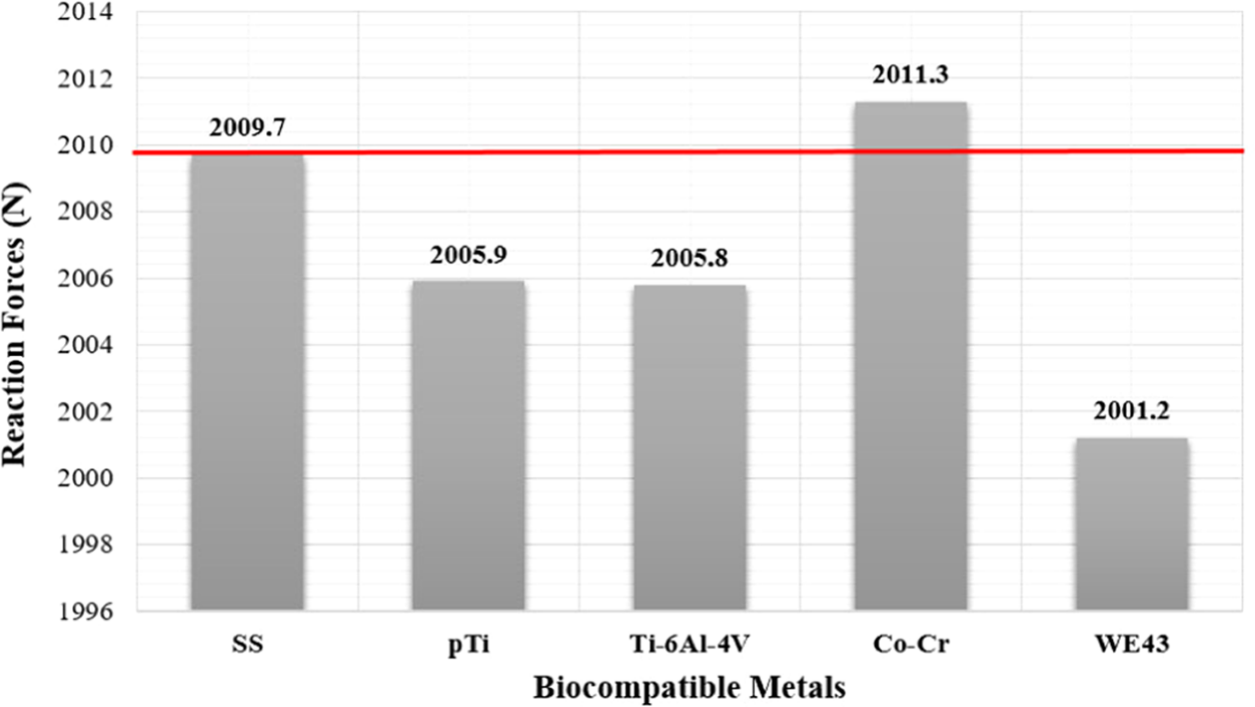
Reaction forces obtained by the static analyses.

To better interpret the reaction forces obtained by static analyses, all values were compared with SS, which is the most preferred implant material for femoral neck fractures. When 1 mm vertical displacement loading was performed, reaction forces on the femur shaft were calculated. The maximum reaction force occurred at the Co–Cr alloy, while the lowest reaction force occurred at the WE43 alloy. As seen in Figure 11, when reaction forces are compared, the reaction force in the Co–Cr alloy is 1.6 N higher than that in SS, whereas the reaction force in the WE43 alloy is 8.5 N less than that in SS.

**Figure 12:**
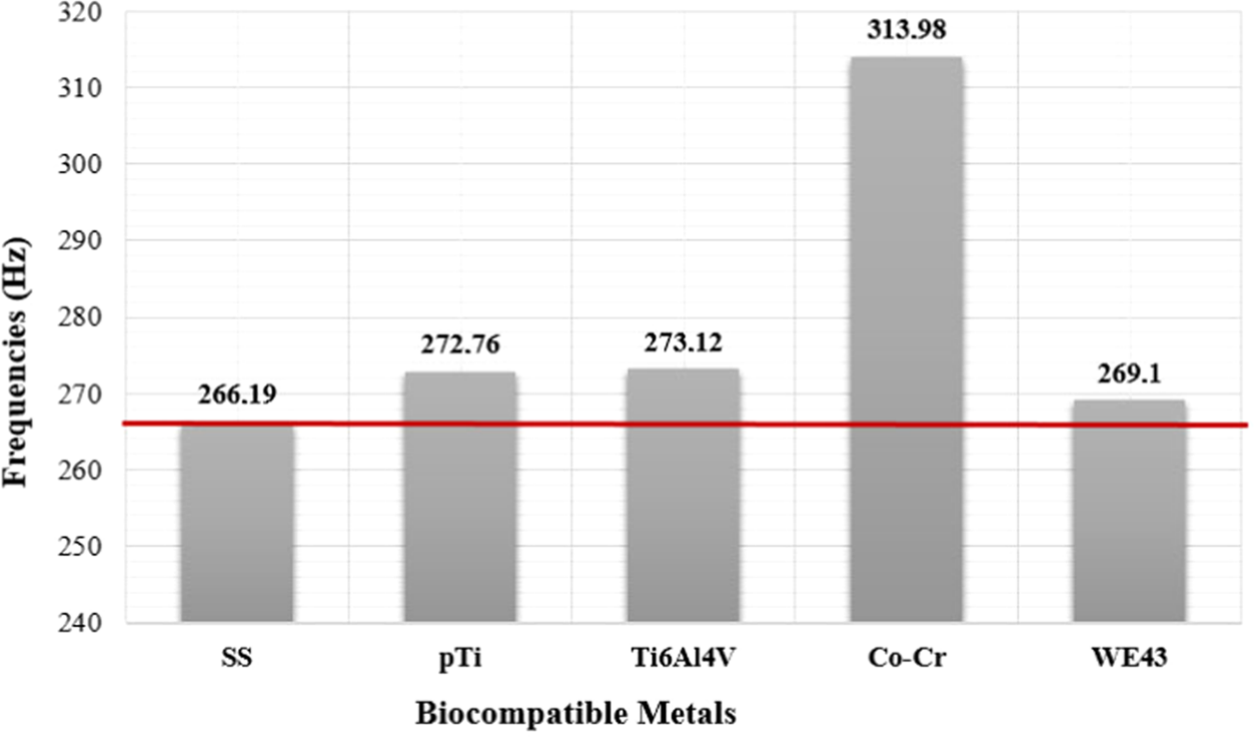
Frequency values obtained by the vibration analyses.

Vibration tests were performed after static analyses. The aim of the vibration analysis, also known as modal analysis, was to observe the dynamic characteristics of the femur. For this purpose, vibration analyses were performed for each material and mode shape and frequency values and period values of the femur were determined. When results were compared with each other, it was determined that mode shapes were the same for all materials. When the frequency values were examined, it was seen that the lowest frequency value occurred in stainless steel with 266.19 Hz and the highest frequency value occurred in the Co–Cr alloy with 313.98 Hz (Figure 12).

**Figure 13:**
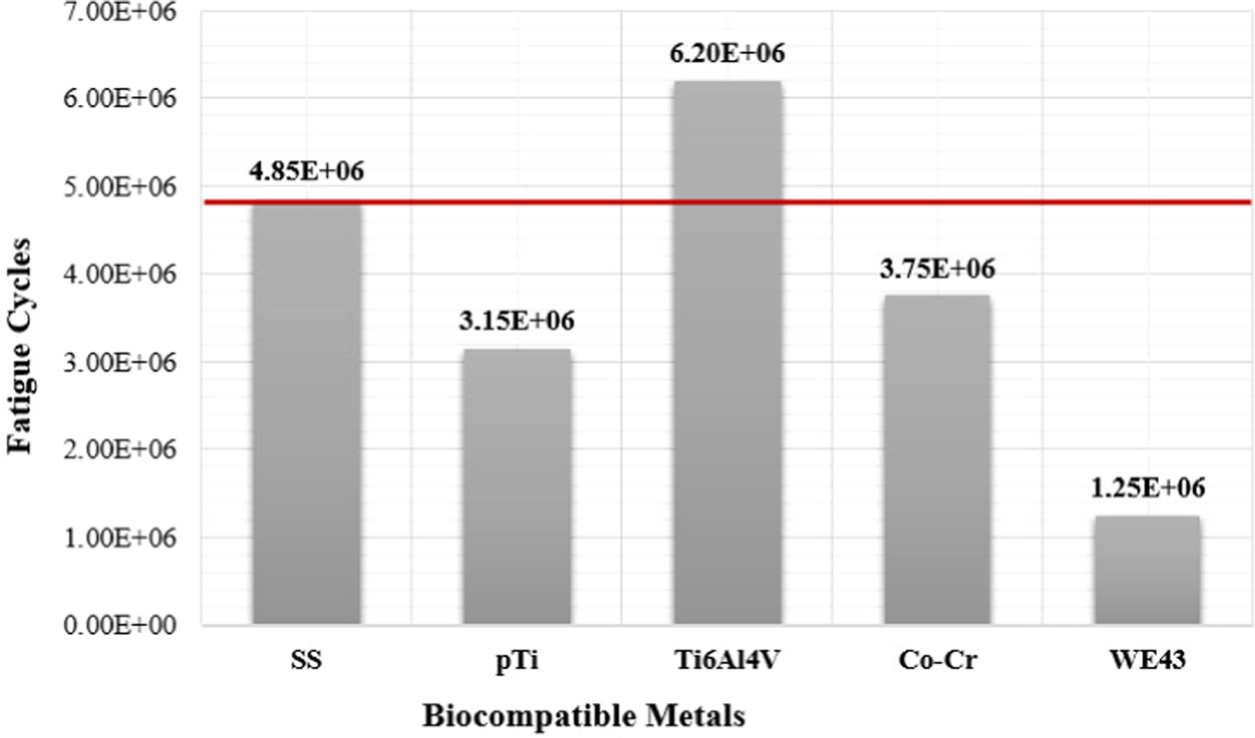
Fatigue lives obtained by the fatigue analyses.

Finally, fatigue analyses were performed for the materials. A vertical 1000 N load was taken into consideration for fatigue analyses. Among the analyses, biocompatible metals were subjected to a periodic and fully reversed loading at a cyclic vertical load of 1000 N. In this study, fatigue cycles of the analyses were examined and the highest fatigue strength was determined for Ti6Al4V and the lowest fatigue strength was determined for the WE43 alloy (Figure 13).

## 5 Identification of the Optimum Material

Engineering science stands essentially on the identification of the best solution for a given problem, which is the equivalent of the optimum design concept. Response to the question “Which one is the best?” requires a performance qualification method after the establishment of significant design criteria.^26,27^ The aim of this study is somehow the solution of an optimization problem searching for the best material to achieve the highest performance for the treatment of an FNF.

Each of the numerical results provides an insight into the behavior of implants made up of different biocompatible materials. Nevertheless, as can be seen throughout the results, there is not a single material that yields the best results on all of the analysis outcomes. For the purpose of overcoming this contradiction, a mathematical model is constituted that could be called an optimality indicator (λ).

This mathematical model is defined as an object function and has the following constraints:
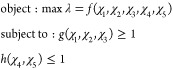
Performance parameters in this optimization problem are Young’s modulus (χ_1_), natural vibration frequency (χ_2_), fatigue life (χ_3_), von Mises stress (χ_4_), and density (χ_5_). For the constitution of the optimality criteria, all of the parameters should be included in the object function one by one while considering the constraints.

Young’s modulus (*E*) is the material property directly related to the stiffness of a solid material. Considering that the treatment of a bone fracture almost never requires displacement of the fracture surface, it is important to use a material that has a high Young’s modulus. Hence, the optimality level based on Young’s modulus value of the *i*th material is first defined.
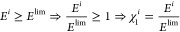
1The second parameter, natural vibration frequency (ω_0_), is required to be higher to decrease the possibility of the resonance. This value is acquired as the first frequency of the femur-implant structure from the finite-element analysis.
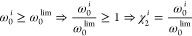
2It is also expected that the third parameter fatigue life (*N*_*f*_) is as great as possible for the implants to contribute to the treatment process without any material failure.
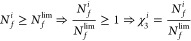
3The fourth parameter is the maximum von Mises stress (σ_vM_) of the implants and is actually calculated based on the maximum principal (σ_1_), minimum principal (σ_3_), and shear (τ) stress values after a finite-element analysis.
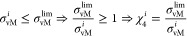
4The density of the materials (ρ) is taken into account as the last parameter while aiming to have a lighter implant.
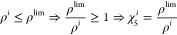
5Limit superior and limit inferior values in the above formulas are the maximum and minimum material property values of SS, pTi, Ti6Al4V, Co–Cr, and WE43. Furthermore, since FEA results reveal that all of the implants have much lower stress values than their material yield strength limits, σ_vM_^lim^ is determined as the minimum value of the performed analyses instead of considering their yield strength values.

Finally, the optimality indicator (λ) is constituted by averaging all of the performance parameters as follows
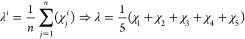
6Application of the numerical results into the formula provides optimality indicator values in Table 7.

**Table 7:** Optimality Indicator Values of the Biocompatible Metals

	SS	pTi	Ti6Al4V	Co–Cr	WE43
**λ**	0.710	0.644	0.741	0.898	0.469

Table 7 shows that the cobalt–chromium alloy (Co–Cr) has the highest value of the optimality indicator (λ) based on its material properties and analysis results. Co–Cr draws the attention owing to the highest values of Young’s modulus, natural frequency, and density and the above-average values of other characteristics. Titanium alloy (Ti6Al4V), having the second-highest λ value, has the longest fatigue life. Although stainless steel (SS) does not have the highest value of any performance parameter, this material ranks third in optimality. It is also seen that the magnesium alloy (WE43) has the last rank owing to its Young’s modulus, critical stress, and fatigue life values.

## 6 Conclusions

Femoral neck fracture (FNF) is a type of injury that is commonly encountered worldwide. Especially osteoporosis or low bone mass is the most important cause of these fractures. The main goal of the treatment of FNFs is to minimize trauma and bring patients back to their prefracture functional levels. Arthroplasty is preferred in the treatment of elder patients, while internal fixation is aimed at young patients. The selection of the treatment technique is primarily based on the type of fracture, patient’s specific medical needs, and risk factors. Today, internal implants are extensively used in the treatment of FNFs. Within implant applications, it is possible to find many different fixation configurations with many different implant materials.

The aim of this study is to investigate the mechanical behavior and effects of the most preferred implant materials on FNFs. In addition, we intended to investigate the effects of material characteristics on implant performance in the application of cannulated screws in an inverted triangle (CSIT), which is mostly preferred by orthopedic surgeons in implant applications. For this purpose, a femur bone with a type 2 fracture was modeled numerically and the performance of CSIT with different materials was investigated via finite-element analysis (FEA).

Within the scope of the research, stainless steel (SS), pure titanium (pTi), titanium alloy (Ti6Al4V), cobalt–chromium alloy (Co–Cr), and magnesium alloy (WE43) materials, which are frequently used in implant applications, were taken into consideration and their performances were evaluated using static analyses, vibration analyses, and fatigue analyses. Additionally, an optimality indicator formula was developed to find the optimum material among these biometals. Throughout the comparison of analysis results, the best material was found to be the Co–Cr alloy on the basis of the considered performance characteristics. In terms of vibration analysis, the highest frequency values were achieved for the Co–Cr alloy along with the highest values of Young’s modulus and material density. However, when fatigue analysis was observed, it was seen that the Ti6Al4V alloy had the highest value, which ensured the second optimality indicator value, although all materials had high cycle fatigue properties.

Considering all of the outcomes of the research, future studies could be constituted and also the identification approach of the material optimality should be improved. Improvement in the formula might be pursuant to the implementation of biocompatibility, corrosion resistance, penetrability, etc. characteristics of the materials.
